# Xanthine oxidase inhibitors treatment or discontinuation effects on mortality: evidence of xanthine oxidase inhibitors withdrawal syndrome

**DOI:** 10.3389/fphar.2023.1289386

**Published:** 2024-01-08

**Authors:** Masanari Kuwabara, Michikazu Nakai, Yoko Sumita, Yoshitaka Iwanaga, Ryusuke Ae, Takahide Kodama, Ichiro Hisatome, Naoyuki Kamatani

**Affiliations:** ^1^ Department of Cardiology, Toranomon Hospital, Tokyo, Japan; ^2^ National Cerebral and Cardiovascular Center, Suita, Japan; ^3^ Division of Public Health, Center for Community Medicine, Jichi Medical University, Shimotsuke, Japan; ^4^ National Hospital Organization, Yonago Medical Center, Yonago, Japan; ^5^ StaGen Co., Ltd., Tokyo, Japan

**Keywords:** xanthine oxidase, xanthine oxidase inhibitors, mortality, withdrawal syndrome, epidemiology, uric acid, hyperuricemia

## Abstract

**Objectives:** This study investigates the impact of xanthine oxidase inhibitors (XOI) on mortality in patients with cardiovascular diseases. XOI withdrawal has been reported to increased mortality risk due to rapid adenosine triphosphate (ATP) deficiency. This study aims to determine whether XOI treatment reduces mortality and whether XOI withdrawal increases mortality.

**Methods:** This is a real-world database study using the Japanese Registry of All Cardiac and Vascular Diseases (J-ROAD). We analyzed 1,648,891 hospitalized patients aged 20–90 with acute coronary syndrome or heart failure. In the first study, mortality rates were compared between patients without urate-lowering agents (n = 1,292,486) and those with XOI agents (n = 315,388, excluding 41,017 on other urate-lowering agents). In the second study, mortality rates were compared between the XOI continuous medication group (n = 226,261) and the XOI withdrawal group (n = 89,127).

**Results:** After multiple adjustments, XOI treatment group showed significantly lower mortality compared with that without any urate-lowering agent (odds ratio (OR), 0.576, 95% confidence interval (CI), 0.567–0.587, *p* < .001). In the sub-analysis, the group with allopurinol (OR, 0.578; 95% CI, 0.557–0.600), febuxostat (OR, 0.610; 95% CI, 0.599–0.622), and topiroxostat (HR, 0.545; 95% CI, 0.473–0.628) showed lower OR of mortality compared with that without any urate-lowering agent. XOI withdrawal group led to significantly higher death rates compared to XOI continuous group (19.8% vs. 0.03%; *p* < .001).

**Conclusion:** XOI treatment for patients with cardiovascular diseases is associated with reduced mortality. Conversely, XOI withdrawal is linked to elevated mortality risk. This emphasizes the importance of both prescribing and discontinuing XOI carefully to optimize patient outcomes.

## Introduction

Uric acid, the end product of adenosine triphosphate (ATP) metabolism in humans, is influenced by xanthine oxidase (XO). XO inhibitors (XOI) suppress the production of uric acid and potentially store ATP (Kuwabara et al., 2023). XOI discontinuation has shown a XOI withdrawal syndrome with ATP depletion and increased mortality (Johnson et al., 2019; Ghang et al., 2020). Cardiovascular Safety of Febuxostat and Allopurinol in Patients with Gout and Cardiovascular Morbidities (CARES) trial showed that febuxostat use is associated with increased cardiovascular-related death compared to allopurinol (White et al., 2018). However, the CARES showed that nearly 85% of deaths occurred while subjects were off of therapy (Bubb, 2019). The CARES sub-analysis found increased major adverse cardiovascular events (MACE) and cardiovascular death verse events were increased in the initial stage after discontinuation of febuxostat or allopurinol (Ghang et al., 2022). The FDA showed black-box warnings for febuxostat (Abeles and Pillinger, 2019), yet the febuxostat versus allopurinol streamlined trial (FAST), which had a low dropout rate, found no group differences in cardiovascular outcomes or death between the two groups (Mackenzie et al., 2020). These results suggest that the primary causes of MACE or death are associated with the withdrawal of XOI regardless of the type of XOI used. We hypothesize that the main cause of death is from XOI withdrawal by removing beneficial effects of XOI like reducing uric acid, reducing reactive oxygen species (ROS) and inflammation, or storing ATP (Feig et al., 2008; Johnson et al., 2019).

This study tests our hypothesis that oral XOI administration improves mortality, but discontinuation leads to excess deaths. This study analyzes inpatient data of acute coronary syndrome (ACS) or heart failure patients, a high-risk population, to compare mortality with and without XOI and XOI continuation and discontinuation. This investigation aims to shed light on the potential benefits of XOI administration and the risks associated with discontinuation in this vulnerable population.

## Materials and methods

### Study design and study subjects

This study retrospectively analyzed the Japanese Registry of All Cardiac and Vascular Diseases (J-ROAD) database, which is a nationwide registry collected by the Japanese Circulation Society (JCS). The database consists of all participating (associated) training hospitals in the JCS (Yasuda et al., 2016; Yasuda et al., 2018). The main diagnoses or comorbidities of each patient were coded using the International Classification of Disease and Related Health Problems 10th revision (ICD-10) codes. As no information specifying individuals was included, the requirement for informed consent was waived. This study complied with the principles of the Declaration of Helsinki regarding investigations in human subjects and was approved by the Toranomon Hospital Institutional Research Ethics Review Board (Approved number 2208).

We collected and analyzed the J-ROAD data from April 2014 to March 2020. The study consists of two studies. As the first study, we compared all-cause mortality in inpatient with ACS or heart failure at admission between with XOI and without any urate-lowering medication. In the sub-analysis of the first study, we checked each XOI (allopurinol, febuxostat, and topiroxostat) affects mortality. As the second study, we compared rate of death between XOI continuous group and withdrawal group.

### Patient involvement

No patients were involved in setting the research question or outcome measures, nor were they involved in the design and implementation of the study. There are no plans to involve patients in dissemination.

### Inclusion and exclusion criteria

**FIGURE 1:**
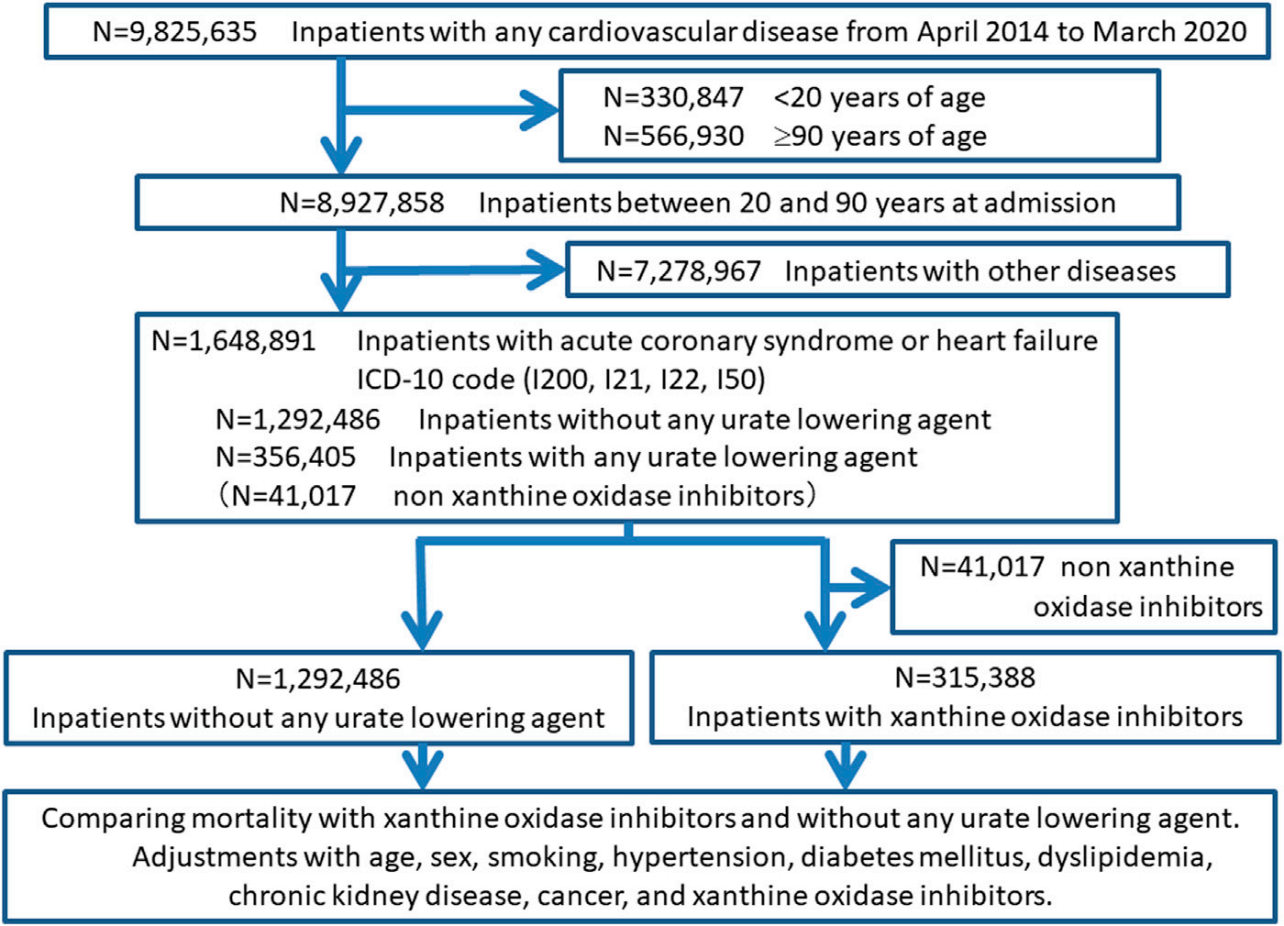
Flow diagram of the first study: xanthine oxidase inhibitors treatment and mortality.

**FIGURE 2:**
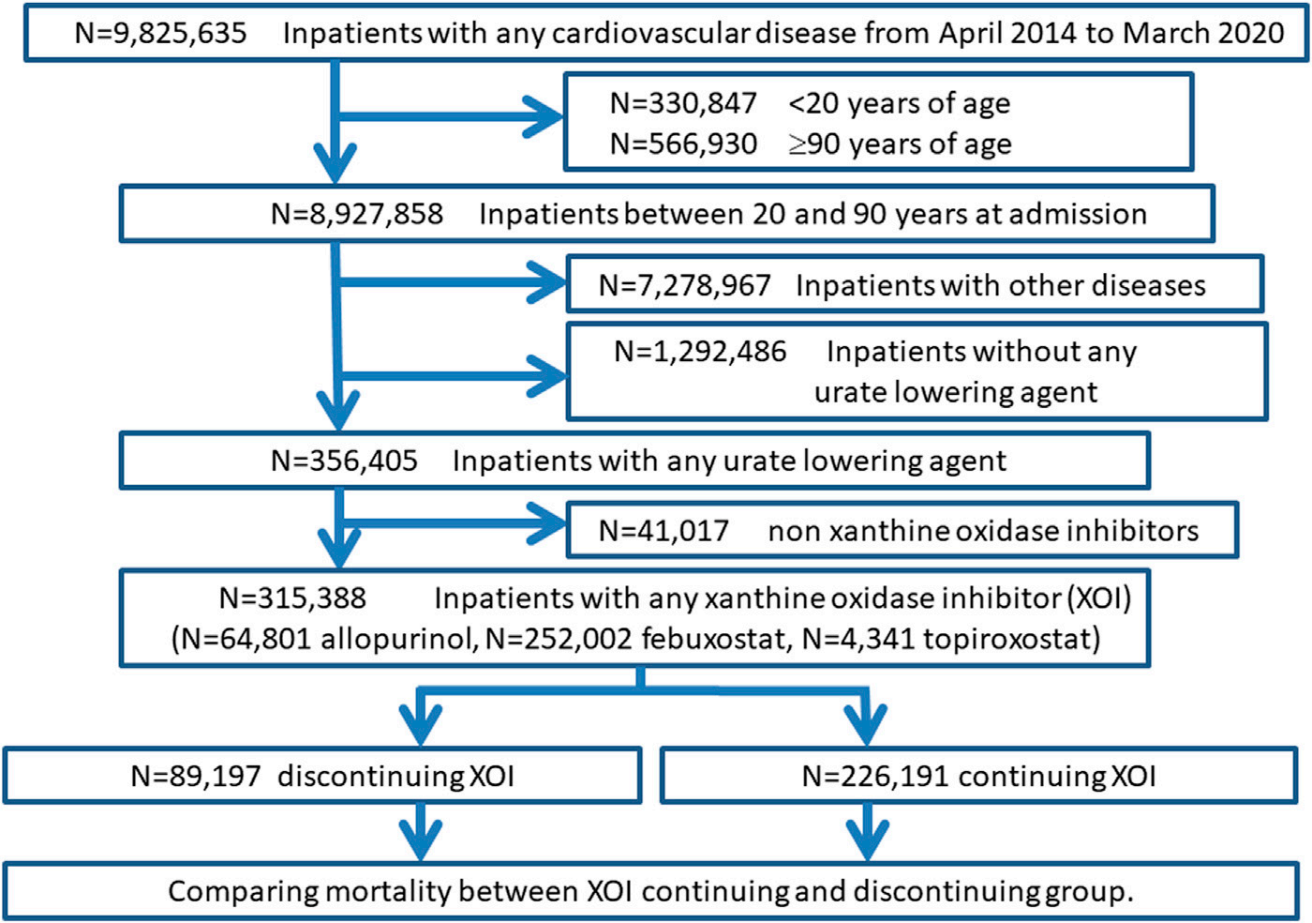
Flow diagram of the second study: xanthine oxidase inhibitors discontinuation and mortality.

The J-ROAD database had 9,825,635 inpatients data from April 2014 to March 2020. We included inpatients aged between 20 and 90 years with ACS or heart failure at admission.

which ICD-10 code was I200, I21, I22, and I50. We showed the flow diagram of the study (Figures 1, 2).

### Statistical analysis

The statistically significant level was set at probability (p) < 0.05 (two sided). Data are expressed as mean ± standard deviation or as percent frequency unless otherwise specified. Comparisons between two groups were performed with student *t*-tests for normally distributed variables, and χ^2^ analyses for categorical data.

In the first study, we compared all-cause mortality between patients with XOI and without any urate-lowering agents. To analyze the factors associated with mortality, a multilevel mixed-effect logistic regression analysis using institution as a random intercept was performed. The factors associated with mortality were evaluated both by crude models (non-adjusted) and by adjusted multivariable models with age, sex, smoking, hypertension, diabetes mellitus, dyslipidemia, chronic kidney disease, cancer, and XOI.

In the second study, we checked mortality by four group categories: 1) those who were on XOI at admission and continued XOI at discharge, 2) those who were on XOI at admission and discontinued XOI at discharge, 3) those who were not on XOI at admission (XOI was given during hospitalization) and continued XOI at discharge, and 4) those were not on XOI at admission (XOI was given during hospitalization) and discontinued XOI at discharge. We compared continuous XOI group including 1) and 3) and withdrawal group including 2) and 4) by χ^2^ analyses and calculate odds ratio.

Statistical analyses were performed using the SPSS Statistics software version 25 for Windows (IBM SPSS Statistics; IBM, New York, USA) and the Stata version 14.2 for Windows (StataCorp, College Station, TX, United States).

### Ethical considerations

We adhered to the principles of the Declaration of Helsinki. Informed consent was obtained from all subjects by a comprehensive agreement method provided by St. Luke’s International Hospital. All data were collected and compiled in a protected computer database. Individual data were anonymous without identifiable personal information. St. Luke’s International Hospital Ethics Committee approved the protocol for this study.

## Results

### Demographics of this study subjects

Of 9,825,635 inpatients data in J-ROAD from April 2014 to March 2020, we included 8,927,858 inpatients out of excluding 330,847 inpatients younger than 20 years and 566,930 inpatients older than 90 years. Of those, we included 1,648,891 inpatients with ACS or heart failure. The backgrounds of the study subjects were shown in Table 1. We divided the inpatients into with urate-lowering agent (N = 356,405) and without any urate-lowering agent (N = 1,292,486). Finally, we compared mortality between 1,292,486 without urate-lowering agent and 315,388 with XOI agent after excluding 41,017 subjects with urate-lowering agent other than XOI.

**TABLE 1:** Backgrounds of the study subjects.

	Total	Women	Men
Number of subjects	1,648,891	587,229	1,061,662
Age (years old)	73.1 ± 11.9	77.2 ± 10.4	70.9 ± 12.2
Smoking	49.4%	20.9%	65.2%
Hypertension	56.3%	55.1%	56.9%
Diabetes mellitus	7.7%	7.0%	8.0%
Dyslipidemia	37.0%	31.6%	40.0%
Hyperuricemia	8.1%	5.9%	9.5%
Chronic kidney disease	13.6%	12.7%	14.1%
Cancer	5.1%	4.4%	5.4%

### Xanthine oxidase inhibitors treatment and mortality

The number of patients with each XOI prescription was following: 64,801 allopurinol, 252,002 febuxostat, and 4,341 topiroxostat. The mortality of the whole patients of this study was 7.6%. The mortality of the study inpatient with allopurinol, febuxostat, and topiroxostat were 5.0%, 5.6%, and 4.8%, respectively, which were significantly lower than that without any urate-lowering agent.

After multivariable adjustments with age, sex, smoking, hypertension, diabetes mellitus, dyslipidemia, chronic kidney disease, and cancer, the group with XOI showed significantly lower odds ratio (OR) of mortality compared with that without any urate-lowering agent (OR, 0.576; 95% CI, 0.567–0.587; *p* < .001). In the sub-analysis, the group with allopurinol (OR, 0.578; 95% CI, 0.557–0.600; *p* < .001), febuxostat (OR, 0.610; 95% CI, 0.599–0.622; *p* < .001), and topiroxostat (HR, 0.545; 95% CI, 0.473–0.628; *p* < .001) showed lower OR of mortality compared with that without any urate-lowering agent (Table 2).

**TABLE 2:** Impact of xanthine oxidase inhibitors on prognosis.

			Crude			Adjusted^a^		
			Odds ratio	95% CI	p	Odds ratio	95% CI	p
Age	Per 1 year increased		1.048	1.047–1.049	<0.001	1.043	1.042–1.042	<0.001
Women	Versus men		1.211	1.197–1.226	<0.001	1.027	1.021–1.041	<0.001
Smoking	Positive versus negative		1.001	0.989–1.013	0.888	1.307	1.289–1.326	<0.001
Hypertension	Positive versus negative		0.225	0.222–0.228	<0.001	0.286	0.282–0.290	<0.001
Diabetes mellitus	Positive versus negative		0.739	0.721–0.757	<0.001	0.772	0.753–0.792	<0.001
Dyslipidemia	Positive versus negative		0.173	0.170–0.176	<0.001	0.266	0.261–0.272	<0.001
Chronic kidney disease	Positive versus negative		1.413	1.391–1.435	<0.001	1.263	1.242–1.284	<0.001
Cancer	Positive versus negative		1.752	1.715–1.791	<0.001	1.216	1.189–1.244	<0.001
XO inhibitors	Positive versus negative		0.661	0.650–0.672	<0.001	0.576	0.567–0.587	<0.001
Allopurinol^b^	Positive versus negative		0.631	0.609–0.654	<0.001	0.578	0.557–0.600	<0.001
Febuxostat^b^	Positive versus negative		0.695	0.683–0.708	<0.001	0.610	0.599–0.622	<0.001
Topiroxostat^b^	Positive versus negative		0.582	0.507–0.669	<0.001	0.545	0.473–0.628	<0.001

XO, xanthine oxidase.

^a^ Data adjusted for age, sex, smoking, hypertension, diabetes mellitus, dyslipidemia, chronic kidney disease, cancer, and XO, inhibitors.

^b^ In the additional analysis, allopurinol, febuxostat, and topiroxostat were included in the analysis instead of XO, inhibitors.

### Xanthine oxidase withdrawal syndrome

Of 356,405 inpatients with any urate-lowering agent, 315,388 were on XOI (41,017 were on other urate-lowering agent). The XOI adherents were divided into the following four groups, and the number of deaths and mortality rates for each were identified as follows;

(1) those who were on XOI at admission and continued XOI at discharge: 219,020 patients, of which 59 deaths (0.03%), (2) those who were on XOI at admission and discontinued XOI at discharge: 89,035 patients, of which 17,663 deaths (19.84%), (3) those who were not on XOI at admission (XOI was given during hospitalization) and continued XOI at discharge: 7,241 patients, of which 11 deaths (0.15%), (4) those were not on XOI at admission (XOI was given during hospitalization) and discontinued XOI at discharge: 92 patients, of which 11 deaths (11.96%).

We compared rate of death between continuous medication group including (1) and (3) and withdrawal group including (2) and (4). The rate of death in XOI withdrawal group was 620 times higher than XOI continuous group (19.8% versus 0.03%; *p* < .001).

## Discussion

This study aimed to assess the prognostic impact of oral administration and discontinuation of XOI in patients hospitalized for ACS or heart failure. The findings revealed that hospitalized patients receiving XOI associated a 42% lower mortality rate compared to those without any urate-lowering agent. This trend was consistent across individual XOIs—allopurinol, febuxostat, and topiroxostat. The results suggest that XOI could reduce mortality regardless of the type of XOI used. Conversely, discontinuation of XOI was associated with a staggering 620-fold increase in mortality when compared to the group that continued XOI therapy. These results substantiate our hypothesis that XOI has intrinsic beneficial effects, while withdrawal negates these effects (Johnson et al., 2019). These results are compatible with some previous studies including the analyses of Medicare data or the United Kingdom Clinical Research Practice Datalink which indicated beneficial effects of allopurinol including reduced mortality in long-term studies (MacIsaac et al., 2016; Singh et al., 2017).

The suggested mechanisms underlying XOI’s cardiovascular benefits encompass the mitigation of negative effects from ROS or hyperuricemia, as well as the positive effects of ATP augmentation (Feig et al., 2008; Kuwabara et al., 2018). The production of uric acid via XO generates ROS like superoxide or hydroxyl radical, which can be harmful to various cells and tissues. Therefore, inhibiting its generation with XOI could alleviate the consequences of ROS production. While uric acid possesses antioxidant properties in serum, it acts as a prooxidant molecule intracellularly. It activates a specific catalytic subunit of nicotinamide-adenine dinucleotide phosphate (NADPH) oxidase, NOX4 (Lanaspa et al., 2012). In the endothelium, uric acid reduces the availability of nitric oxide, a vasodilator, leading to vascular endothelial damage and dysfunction, which contributes to the development of cardiovascular diseases (Kuwabara et al., 2018). While the ROS mechanisms require longer durations to manifest, Noman et al. demonstrated the prompt benefits of allopurinol over a mere 6-week period in stable chronic angina patients (Noman et al., 2010). This implies that XOI’s positive impact becomes evident rapidly, and withdrawal promptly reverses these effects. ATP directly affects many organs including heart and its effects are rapid. Upon XOI cessation, the avoided deaths reappear by the lack of ATP particularly impacting the heart, leading to increased death in at-risk individuals. The notion of a “XOI withdrawal syndrome” is similar to withdrawal effects observed with cardiovascular risk-reducing medications like beta-blockers (Prins et al., 2015), a cornerstone in treatment for heart failure with reduced ejection fraction (McDonagh et al., 2021; Tsutsui et al., 2021; Heidenreich et al., 2022).

**FIGURE 3:**
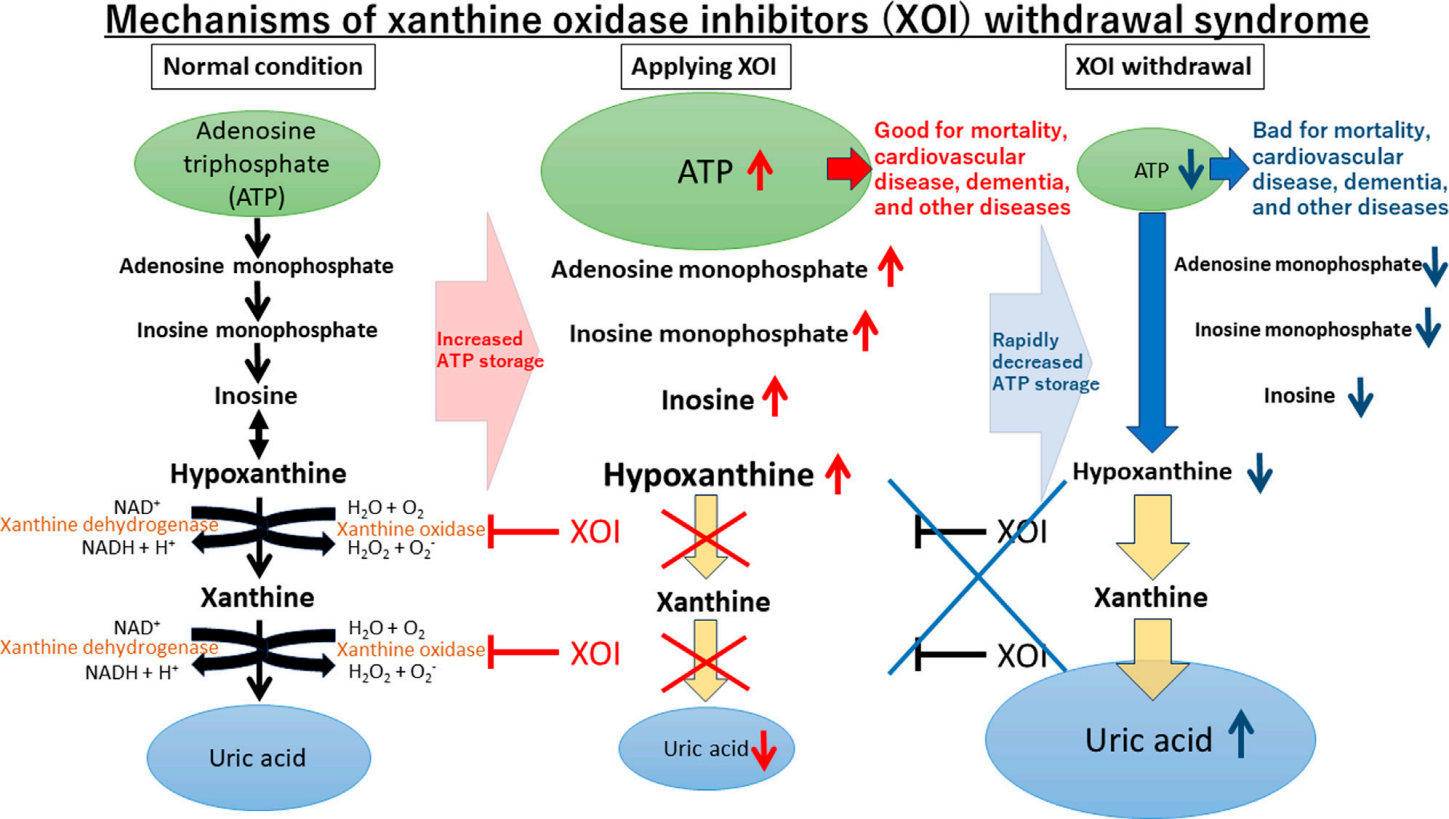
Mechanisms of xanthine oxidase inhibitors (XOI) withdrawal syndrome.

Although this study assessed all-cause mortality, detailed cause-specific mortality data were constrained by data limitations. Notably, dementia is a major contributor to poor mortality (Wolfson et al., 2001; Mitchell, 2015). Recent studies indicated a potential association between gout or hyperuricemia and dementia, suggesting higher uric acid levels was associate with lower prevalence of dementia (Scheepers et al., 2019; Min et al., 2021). Conversely, a meta-analysis unveiled a negative relationship between allopurinol exposure and dementia risk (Lai et al., 2022), implying allopurinol’s protective effects. To delve into this, we undertook additional analyses to investigate XOI’s potential link to dementia. The results demonstrated that the XOI group exhibited a significantly lower OR of dementia compared to the group without any urate-lowering agent, even after accounting for various factors (OR, 0.893; 95% CI, 0.874–0.912; *p* < 0.001) (Table 3). In the subgroup analysis, allopurinol and febuxostat demonstrated a similar trend, whereas topiroxostat did not attain statistical significance. Our results align with recent studies indicating that allopurinol and febuxostat were associated with a reduced risk of developing Alzheimer’s disease or dementia (Singh and Cleveland, 2018; Fang et al., 2022). These findings lend support to our hypothesis suggesting that XOI might potentially contribute to dementia reduction through ATP storage (Figure 3).

**TABLE 3:** Impact of xanthine oxidase inhibitors on dementia.

			Crude			Adjusted^a^		
			Odds ratio	95% CI	p	Odds ratio	95% CI	p
Age	Per 1 year increased		1.149	1.148–1.151	<0.001	1.136	1.134–1.138	<0.001
Women	Versus men		2.516	2.475–2.558	<0.001	1.462	1.440–1.496	<0.001
Smoking	Positive versus negative		0.472	0.463–0.480	<0.001	0.910	0.892–0.929	<0.001
Hypertension	Positive versus negative		0.733	0.721–0.746	<0.001	0.83	0.813–0.842	<0.001
Diabetes mellitus	Positive versus negative		0.661	0.639–0.685	<0.001	0.878	0.895–0.962	<0.001
Dyslipidemia	Positive versus negative		0.407	0.399–0.416	<0.001	0.624	0.607–0.635	<0.001
Chronic kidney disease	Positive versus negative		1.021	0.998–1.045	<0.001	0.92	0.896–0.941	<0.001
Cancer	Positive versus negative		0.942	0.908–0.978	<0.001	0.748	0.722–0.779	<0.001
XO inhibitors	Positive versus negative		0.988	0.968–1.009	<0.001	0.895	0.874–0.912	<0.001
Allopurinol	Positive versus negative		0.82	0.784–0.858	<0.001	0.789	0.754–0.826	<0.001
Febuxostat	Positive versus negative		1.037	1.014–1.060	<0.001	0.931	0.909–0.953	<0.001
Topiroxostat	Positive versus negative		1.163	1.009–1.342	0.038	1.134	0.980–1.314	0.092

XO, xanthine oxidase.

^a^ Data adjusted for age, sex, smoking, hypertension, diabetes mellitus, dyslipidemia, chronic kidney disease, cancer, and XO, inhibitors.

^b^ In the additional analysis, allopurinol, febuxostat, and topiroxostat were included in the analysis instead of XO, inhibitors.

Allopurinol versus usual care in UK patients with ischaemic heart disease (ALL-HEART) study showed that treatment with allopurinol did not affect the risk of cardiovascular outcomes (Mackenzie et al., 2022). In the ALL-HEART study, the serum uric acid level in the allopurinol group was 0.34 mmol/L (5.7 mg/dL) at baseline and decreased to 0.18 mmol/L (3.0 mg/dL) after treatment. However, this study did not specifically assess the effects of hyperuricemia treatment due to its design. Additionally, the study experienced a high dropout rate, with 57.4% of participants in the allopurinol group withdrawing from the randomized treatment. The CARES trials showed that all-cause mortality and cardiovascular death was higher in febuxostat group compared with allopurinol group, (White et al., 2018), but a *post hoc* analysis revealed most deaths in the drug discontinuation group (Johnson et al., 2019). Given the results of our study, it raises the possibility that there were more cardiovascular events in the group that withdrew allopurinol and fewer cardiovascular events in the group that continued allopurinol in the ALL-HEART study. Further analyses comparing the outcomes between the groups that continued and those that withdrew from the treatment are necessary. We hypothesize that in the ALL-HEART study, the group that continued medication may have experienced a reduction in events, while the discontinuation group saw an increase, leading to an overall non-significant difference in outcomes.

Our study has several limitations. First, we could not assess uric acid levels and a history of gouts from this database. Recently, a study showed that gout flares are associated with a transient increase in cardiovascular events especially within 120 days after gout flares (Cipolletta et al., 2022). The rate of gout in patients with XOI should be higher than those without any urate-lowering agent, and these groups were higher risk of cardiovascular disease. However, the better prognosis in the XOI group is a noteworthy result. Second, some of the deaths in the XOI discontinuation group were due to the fact that other medications were also discontinued, which indicates it is impossible to determine a direct causal relationship between XOI withdrawal and death. In addition, it is influenced by the fact that some patients tend to stop taking their medications prior to death. Therefore, we may overestimate the effects of XOI withdrawal syndrome. However, there are similar reports about XOI withdrawal syndrome, (Bubb, 2019; Johnson et al., 2019; Ghang et al., 2020; Ghang et al., 2022), and we should take care of discontinuing XOI. Third, we did not assess the impact of other medications, including those for ACS and heart failure. These medications can influence mortality, representing a limitation of this study. However, XOI has minimal confounding effects with other medicines, and we believe that our results remain reasonably acceptable. Fourth, our study has shown favorable outcomes in patients with hypertension, diabetes, and dyslipidemia, which may appear unreasonable. However, considering that the study specifically focused on patients hospitalized for cardiovascular diseases, these positive prognostic results could be attributed to the proper treatment provided to these patients. Fifth, due to the nature of this being a retrospective database study, we have been unable to obtain detailed information on the reasons for the initiation or discontinuation of medications and specific information on cardiovascular deaths. Previous studies have shown that XOI is often prescribed for asymptomatic hyperuricemia rather than for gout in Japan (Hakoda and Kasagi, 2019). Therefore, we assume that in this study, XOI was frequently used for treating asymptomatic hyperuricemia. Although detailed information on cardiovascular deaths would have been more informative, the available data on all-cause mortality still provide valuable insights into the XOI withdrawal syndrome observed in this study. Sixth, this study included patients admitted with ACS or heart failure, recognizing that many cases were complex and involved multiple conditions. Although a sub-analysis for each disease would be valuable, the complexity and overlapping nature of these diseases often make isolated analysis difficult in this database. Further research focused on individual diseases is needed. Finally, it is important to acknowledge that our study is limited in its scope, as it exclusively evaluated Japanese population. To assess generalizability, similar studies should be conducted with other populations. In Japan, insurance covers urate-lowering drugs for hyperuricemia, and the concept of treatment for hyperuricemia may differ from that in other countries.

In conclusion, our study found that inpatients with ACS or heart failure receiving XOI treatment exhibited favorable mortality outcomes. On the other hand, XOI withdrawal was associated with a significantly higher risk of death, indicating the presence of XOI withdrawal syndrome. These findings emphasize the importance of not only prescribing XOI medication but also carefully considering the withdrawal process to optimize patient outcomes.

## Data Availability

The data analyzed in this study is subject to the following licenses/restrictions: The data underlying this article were provided by the Japanese Circulation Society by permission. Data will be shared on request to the corresponding author with permission of the Japanese Circulation Society. Requests to access these datasets should be directed to J-ROAD Secretariat, dpc-jroad@ml.ncvc.go.jp.
